# The Use of Starch Drying Kinetics Curves for Experimental Determination of Its Specific Surface Area

**DOI:** 10.3390/molecules26185508

**Published:** 2021-09-10

**Authors:** Marzena Włodarczyk-Stasiak, Artur Mazurek

**Affiliations:** Department of Analysis and Evaluation of Food Quality, Faculty of Food Science and Biotechnology, University of Life Sciences in Lublin, Skromna Street 8, 20-704 Lublin, Poland; artur.mazurek@up.lublin.pl

**Keywords:** surface area, starch, drying, water vapour sorption

## Abstract

The most popular method for the calculation of specific surface area is its determination from water vapour sorption isotherms. The study presented here has been designed for the purpose of optimisation and selection of the conditions of drying so as to allow the determination of specific surface area from plotted curves of the drying process. The results indicate that drying curves can be used as the basis for the determination of specific surface area, the values of which do not differ statistically significantly (α = 0.05) from those determined from isotherms of water vapour sorption (adsorption/desorption).

## 1. Introduction

The structure of food in an unprocessed state and that formed as a result of technological processes is the object of interest of numerous researchers, as it affects such properties of the product such as its quality, shelf life, nutritional value, functionality, and possibility of unconventional use [1,2,3,4]. 

The surface of food products with features of a solid body is referred to as heterogeneous, which results from the chemical composition and structure of the products. The heterogeneity of the surface of a food product differentiates it in terms of energy and of the appearance of adsorption sites with various levels of activity [5].

The analysis of the structure of a product is conducted most frequently with the use of sorption methods. Measurements are made in the process of adsorption and desorption, from the gaseous phase to the liquid phase, determining the sorption isotherms. Mathematical interpretation of their shapes and knowledge of sorption theories allows the determination of parameters such as monolayer capacity (a_m_) and specific surface area (S_BET_).

At the various stages (production, storage, preparation, consumption), food is in permanent contact with water vapour in the surrounding air. The use of water in its gaseous phase as the adsorbate allows the characterisation of the structure of the material and the theoretical description of physical phenomena taking place on the surface of the system of food–volatile substances (water vapour). Knowledge of the run of the processes of water vapour sorption (adsorption and desorption) finds a practical application in the prediction of the shelf life of food products, assessing their sensitivity to moisture or their susceptibility to drying out.

The monolayer capacity or value (a_m_) is an important parameter determined from the water vapour sorption isotherms. That parameter defines the state in which the surface of a product is saturated with a single layer of water vapour molecules, after exceeding which the excess of water contributes to undesirable changes in the product, such as clogging, hardening, loss of flavour or the appearance of foreign flavours and tastes, microbial growth, which corresponds to the critical moisture level [6]. The critical moisture level is a relative value, dependent on the kind and range of changes that we wish to prevent. Knowledge of the capacity of the molecular layer is an important parameter in the choice and design of packaging in terms of water vapour permeability, in the selection of food storage conditions and times, in the determination of the safe level of moisture in multicomponent food systems, in the prediction of the run and of the energy consumption for drying processes or for compaction [7,8]. Knowledge of the run of the process of water vapour desorption for a dried product allows for the determination of the endpoint of drying, as an important parameter both for the attainment of the safe moisture of the product and in relation to the energy requirements of the process [3,4,6,9,10,11,12].

However, sorption methods based on experimental determination of water vapour sorption isotherms (adsorption and/or desorption), determination of the monolayer value (a_m_) and calculation of specific surface area from the BET theory (S_BET_), are relatively labour- and time-consuming. It assumes the attainment of equilibrium moisture of the analysed product at a minimum of ten levels of relative humidity of the environment. In practice, this requires multiple weighing of samples, often over periods longer than a month. This stimulated the search for simpler research methods that will minimise the duration of measurements and allow for the determination of the status of water in the product and identification of the mechanisms determining the sorption. A safe level of moisture (critical moisture) is a state in which the material surface is coated with a monolayer of water molecules (monolayer value). From the curve showing the drying progress, it is possible to determine the said critical moisture content. By convention, three drying stages are assumed: Stage I—the period of heating the product to the temperature at which water is removed from the product and evaporated until the critical moisture is reached and the water loss is insignificant; Stage II—the period from reaching the critical moisture until the product reaches equilibrium moisture. At this stage, the dried product reaches a moisture close to that of the drying agent. The period of constant drying rate; Stage III—the period of falling drying speed. Modelling of the drying curve I derivative allows determining the greatest loss of water vapour between successive measurements, which corresponds to the value of the critical moisture. The objective of the study presented herein is the optimisation and selection of drying parameters with the use of drying curves for the determination of the specific surface area.

## 2. Results

**Figure 1:**
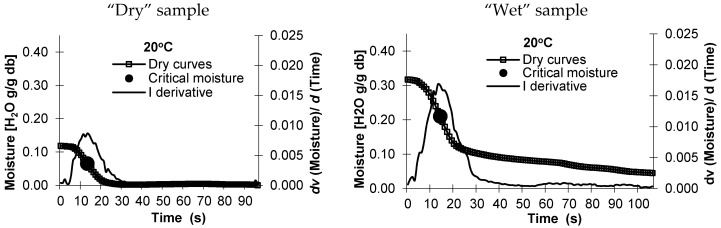

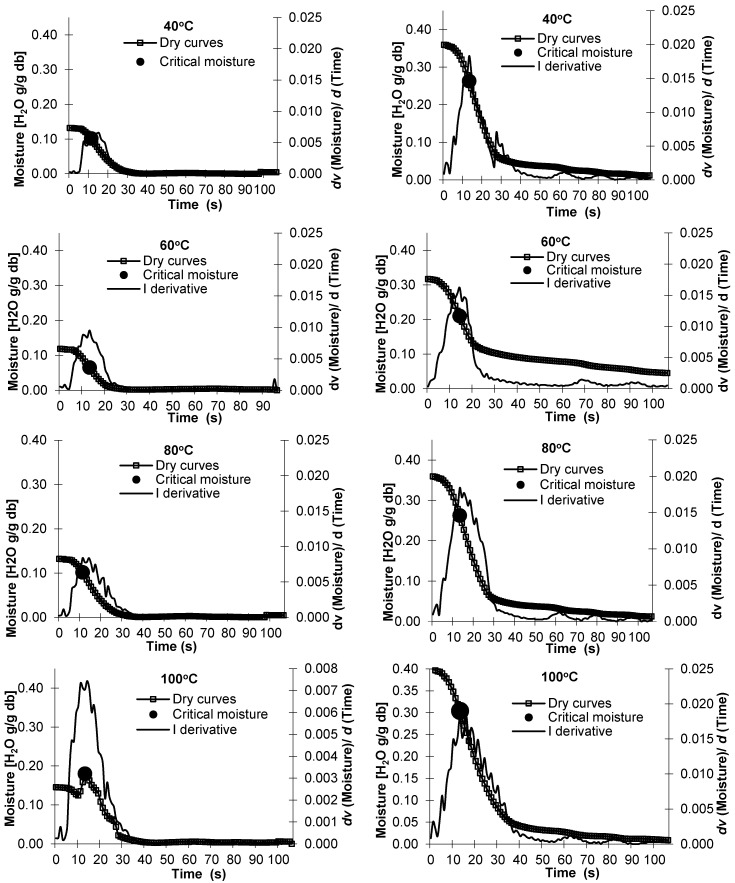
The course drying curves of the “dry” and “wet” potato starch, at a temperature of 20 °C, 40 °C, 60 °C, 80 °C, and 100 °C.

The specific surface area was determined for native starches and for starches modified with various methods. The specific surface area was determined from sorption isotherms (adsorption and desorption) and from the drying curves (at five levels of temperature). Due to the limited volume of the work, 10 out of 90 charts are presented for the selected starch–potato (Figure 1). The data were processed with the use of ANOVA, with the significance level set at α = 0.05.

In the first stage of the data analysis, it was tested whether the obtained values of the specific surface area (S_BET_) determined from water vapour sorption isotherms differ statistically (α = 0.05) from the values calculated from the drying curves for the “dry” and “wet” samples (Table 1). It was adopted that the determination of the specific surface area from the sorption isotherms is a standard method, generally accepted among researchers.

All values of the specific surface area determined from the drying curves for dry samples differed statistically significantly from the values of S_BET_ calculated from the water vapour sorption isotherms.

In the next stage of testing, it was verified whether there were statistically significant differences (α = 0.05) between the values of the specific surface area determined from the adsorption and desorption isotherms and those determined for “wet” samples from the drying curves (Table 2).

Statistical analysis of the significance of differences indicates good assumptions of the experiment, as for a majority of starch samples the values of the specific surface area (S_BET_) do not differ statistically significantly (α = 0.05), irrespective of the process (adsorption/desorption) or of the analytical method (sorption of water vapour/drying). However, a relatively large scatter of results was noted (*x* − 2σ; *x* + 2σ) in the case of S_BET_ determination in the process of drying. Those results related to five temperature levels during drying, which was the probable cause of such a scatter, and therefore another variable was introduced in subsequent comparisons, i.e., the level of temperature during drying (Table 3 and Table 4).

It was tested whether there exist statistically significant differences (α = 0.05) between the value of specific surface area determined from the sorption isotherms (adsorption—Table 3; desorption—Table 4) and the five adopted levels of drying temperature (20 °C, 40 °C, 60 °C, 80 °C and 100 °C) for “wet” samples.

At the temperature levels of 80 °C and 100 °C, statistically significant differences in the value of specific surface area were noted for most of the samples. At those drying temperatures, S_BET_ determined from the drying curves is lower than the values determined from the sorption isotherms by from several to 50%.

Lowering of the drying temperature to 60 °C caused that the differences in the values of specific surface area determined from the isotherms of water vapour adsorption and desorption, and those obtained from the drying curves for four samples (PS, RS, TS, and KS) were not statistically significant (Table 3 and Table 4). Further lowering of the drying temperature to 40 °C did not cause any satisfactory effect on the fitting of the two compared methods of determination of specific surface area.

A fairly good fit of the proposed models used for the determination of specific surface area at the drying temperature of 20 °C was noted. Statistical analysis demonstrated that at the adopted significance level of α = 0.05 no statistically significant differences were found for eight out of nine analysed samples. Such a good level of fit was noted both for the model based on specific surface area (S_BET_) determination from the adsorption isotherm vs. drying curve at the temperature of 20 °C, and analogously, S_BET desorption_ vs. drying curve, 20 °C (Table 5).

## 3. Discussion

The specific surface area is an important property informing about the degree of development of the surface of a material, which affects the run of surface phenomena such as, e.g., sorption (adsorption and desorption). The key role in those processes is that of the number and quality of adsorption centres, and their availability. 

The most common kind of sorption in the technology of food processing is the adsorption and desorption of water vapour. The staling of food products whose moisture is higher than that of the environment, generally considered to be unfavourable, takes place through desorption. That process can also have a beneficial effect, during the preservation of food products through drying. In a situation where the moisture of the environment is higher than that of a product, we are dealing with moisture (water vapour) adsorption by food products. This process is most frequently of a negative character, as it contributes to the clogging and wetting of products, and also causes the increase in moisture level above the critical value, i.e., the state in which a product is no longer susceptible to physicochemical transformations with the participation of water. In the physical aspect, critical moisture corresponds to the amount of water in the monomolecular layer. The BET theory defines this as the monolayer capacity or value, i.e., surface saturation with a single layer of water vapour molecules, permanently bound by adsorption centres. The larger the number of active centres on a developed surface, the greater the amount of water vapour molecules adsorbed in the monomolecular layer. From a technological point of view, this indicates a higher level of critical moisture, i.e., greater stability of a product with a relatively higher moisture. Mathematical interpretation of the monolayer value (mg/g), mass of water molecule (18 g/mol) and surface area of the vertical projection of water molecule (settlement area) allows the calculation of specific surface area (S_BET_).

At the first stage of the research, it was tested whether the moisture level of the samples is an important factor in determining the specific surface area (S_BET_) from the drying curves. The presented results (Table 1) indicate statistically significant differences (α = 0.05) determined from the drying curves for dry samples that differed statistically significantly from the values of S_BET_ calculated from the water vapour sorption isotherms.

It can be assumed that in the course of drying, all unbound water was removed from the samples not only from the surface areas of key importance in the determination of the specific surface area (S_BET_), hence such high values of the specific surface area. Statistical analysis indicates that samples of wet starch, whose specific surface area was determined from the drying curves, and of starch samples for which the values of S_BET_ were determined from sorption isotherms, form homogeneous groups. This indicates, therefore, that the differences in the obtained values of specific surface area are not statistically significantly different, at the adopted significance level of α = 0.05. The wetting of the samples caused that the first to be removed was water in the form of vapour, from the sample surface, i.e., areas where adsorption centres are situated, which play a key role in the determination of specific surface area with the classic method of water vapour sorption. Therefore, it was assumed that in the case of samples defined as “dry”, the state of water in the material and the mechanism of changes taking place as a result of drying are fundamentally different than in the processes of water vapour sorption. On that basis, and with significant statistical differences (α = 0.05) in the obtained values of specific surface area, it was decided to reject the batch of “dry” samples in further testing.

It is known that the state of equilibrium can be attained through adsorption but also through desorption. Therefore, it was analyzed whether there were statistical differences (α = 0.05) between the specific surface area determined from the adsorption and desorption isotherm and the surfaces determined for the “wet” samples from the drying curves (Table 2). Research by Sokołowska [13] indicates that the determination of specific surface area through desorption is burdened with a smaller error than is the case with determination through adsorption. This is attributed to the fact of complete emptying of pores of the adsorbate during desorption, which is not always achievable during sample preparation for the determination of specific surface area from adsorption isotherms. Incomplete pore emptying may result in a reduction in the number and availability of adsorption centres and, consequently, lower values of specific surface area determined through adsorption compared to desorption (S_BET adsorption_ < S_BET desorption_).

In subsequent stages of the experiment, the influence of temperature during drying (20 °C, 40 °C, 60 °C, 80 °C and 100 °C) was analyzed for wet samples on the actual surface value.

At temperatures of 80 °C and 100 °C, for most of the samples, there were statistically significant differences in the value of the specific surface area (Table 3 and Table 4). As a result of sample wetting, the availability of water was increased, but the level of the wetting was low (20–27%) enough not to allow full range gelatinisation. Olu-Owolabi [14] discuss the lack of pasting, preservation of the granular structure and slight changes in functional properties. The authors [14] carried out a hydrothermal modification of bean starch at moisture levels of 20%, 25% and 30%, heated in an air oven at 100 °C. The probable cause of such low values of SBET from the drying curves should be attributed to the formation of a coat/film from damaged starch granules and to the effect of high temperature, which hinders water evaporation in the course of drying.

Lowering the temperature to 60 °C and 40 °C shows a statistically significant difference (α = 0.05) between the compared methods. The reason for this at a drying temperature of 40 °C can be potentially attributed to two processes—swelling and glass transition. It is a well-known fact that the temperature of glass transition (Tg) depends on the content of water in the material. As follows from the Gordon–Taylor equation, the Tg temperature decreases with an increase in the degree of wetting of the material [15]. It can, therefore, be concluded that in amorphous regions of wetted starch granules (also damaged ones) there take place changes that cause an expansion of areas with crystalline properties, which causes changes in the quality and number of sites of key importance in the process of sorption. This assumption is questionable in view of the study by [16] who argue that at 30 °C, 40 °C, 50 °C and starch sample wetting below 60% (*w*/*w*) there are no visible changes in starch that would indicate its gelatinisation. However, the conditions of our study and the mechanism of drying consisting of the extraction of moisture from the material, counteracting the energy retaining moisture [8], make it difficult to explain from the current state of research. The literature provides a fairly large number of reports on the hydrothermal conditions in which the process of swelling can be initiated [17,18]. Irrespective of the botanical origin of starch, according to the cited authors, in a temperature range from 30 °C to 50 °C and water availability (50:50), with an increase in dynamic viscosity, a three-dimensional network is formed from swollen starch granules and linear amylose chains. Although the formed 3D structure could cause an increase in the availability of new sites for water vapour molecules (during adsorption) [17] or facilitate water vapour removal (during desorption), at starch sample moisture levels of 20–27% the conditions allowing starch granule swelling are not met. 

Due to the adopted drying temperature conditions (20 °C), and those prevailing in the course of the classic determination of sorption isotherms, the mechanism of migration of water vapour molecules is similar. In the classical static-desiccator method, the state of equilibrium is attained through water vapour adsorption/desorption by a sample relative to the relative humidity of the environment, regulated by the use of sulphuric acid at various concentrations. During drying, the state of equilibrium moisture of samples is established through desorption. Relative air humidity around the heating element of the moisture analyser is significantly lower than air humidity at the boundary of the sample. During the drying of a wetted sample, diffusion leads to the migration of water vapour, initially only from the surface of the sample, and with the progressing process of drying—also from deeper areas in the sample, until an equilibrium between the drying medium and the sample is reached, i.e., until the equilibrium moisture of the sample is attained. 

Based on the conducted tests and statistical analyses, we can conclude that the suitable choice of the drying conditions allows the obtainment of satisfactory values of specific surface area, comparable to those determined from water vapour sorption isotherms. The experiment presented herein requires further research on heterogeneous materials such as food materials and products. 

## 4. Materials and Methods

### 4.1. Materials 

The material used in the study consisted of native starches of diverse botanical origin and modified maize and tapioca starches (Table 6). The use of homogeneous material in the pilot study should significantly facilitate the interpretation of results.

#### 4.1.1. Preparation of Starch Samples

Starch samples used in the study were of output moisture (Table 7) and were referred to in this text as “dry”. The second group of samples consisted of the same starch samples that had been moistened and those were referred to as ‘wet”. Sample moistening was conducted by placing the samples in a chamber with a relative air humidity of 100%, which corresponded to a_w_ = 1. Starch material was kept in the chamber until the state of equilibrium moisture had stabilized, monitoring the changes with the use of a balance with an accuracy of 0.0001 g. Tymol was placed in the chamber to prevent the growth of microflora.

### 4.2. Methods

#### 4.2.1. Water Vapour Sorption

**Figure 2:**
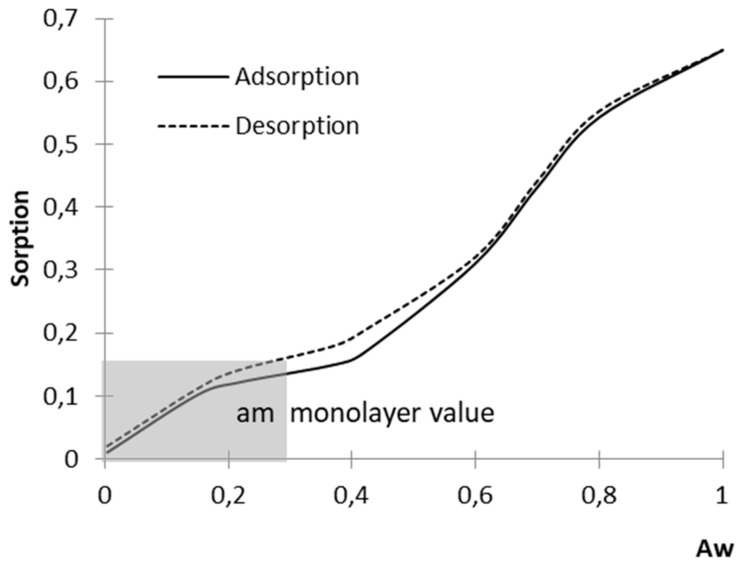
The course of water vapour sorption curves for native starch.

Water vapour sorption measurements of the samples (2 g) were taken at 20 °C. Air humidity control was effected by means of a water solution of sulphuric acid serving for the determination of a_w_ [22]. The obtained experimental data from water vapour sorption were modelled using the BET equation. The monolayer value (a_m_) was calculated from a linearized form of the BET Equation (1), on the basis of monomolecular adsorption of water vapour within the water activity (a_w_) range of ~0.01–0.35 (Figure 2).

a_m_ = 1·(tg α + a)^−1^ (1)

where a_m_–monolayer value [g H_2_O/g db], a–adsorption [g/g db].

Specific surface area was determined from Equation (2) [10,23].

S_BET_ = (a_m_ · σ_o_ · N_o_)·m^−1^ (2)

where S_BET_—specific surface area [m^2^·g^−1^], a_m_—monolayer value [g H_2_O/dbg], σ_o_—settlement area of a molecule of water [10.8 × 10^−20^ m^2^·molecule^−1^], N_o_—Avogadro’s number [6.023 × 10^23^], and m—molecular weight of water [18·gmol^−1^]

#### 4.2.2. Drying Curves

Drying curves were determined with the use of a moisture balance recording the loss of moisture with an accuracy of 0.0001 g, at 1 s intervals (RadWag). The measurements were taken on 2 g samples, using the so-called standard profile (which guarantees rapid attainment of the required temperature), at five temperature levels—20 °C, 40 °C, 60 °C, 80 °C and 100 °C. The drying curves were determined for samples referred to conventionally as “dry” and “wet” (Section 4.1.1). The first derivative method was applied for the calculation of moisture changes of the analysed samples in time (3).

M_t_ = ∆M·∆t^−1^ (3)

where M_t_—first derivative of moisture vs. t [(g H_2_O/1 g db)·t^−1^], ∆M—changes in moisture level-the difference between two consecutive measurements, M_n_–M_n−1_ [g], ∆t—time difference between two consecutive measurements, t_n_–t_n−1_ [s]

**Figure 3:**
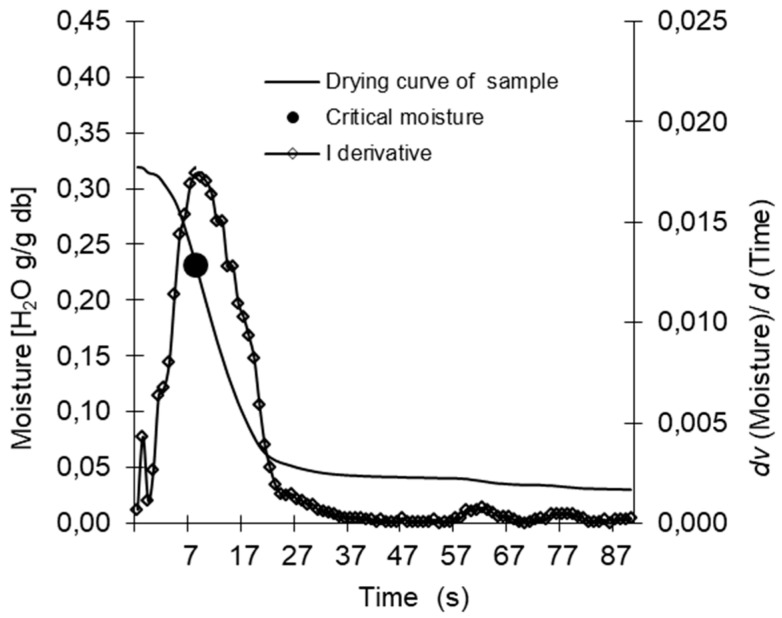
The course of drying sorption curves for native starch.

Mathematical interpretation of the run of the process of drying allows the determination of the critical moisture (M_c_) from the following relation (4) (Figure 3):

M_c_ = *max* M_t_ (4)

where M_c_—critical moisture [g H_2_O/1 g db].

Assuming that the safe level of moisture (critical moisture) is the state in which the surface of the material is covered with a single layer of water molecules (monolayer capacity), Equation (2) was used to calculate the specific surface area.

### 4.3. Statistical Analysis

The data reported in all the results are an average of triplicate observations. In the study, the mean values (*x*) and the standard deviation (σ) were calculated from the range (*x* − 2σ; *x* + 2σ); data out of the range were rejected. The ANOVA was used to calculate significant differences in treatment means and LSD (*p* < 0.05).

## 5. Conclusions

The results indicate that there is a possibility of such optimisation and selection of drying parameters that will allow the use of the curves of the drying process for the determination of specific surface area of starch. The initial moisture of starch proved to be a key parameter affecting the values of specific surface area. The wetting of samples prior to the determination of their drying curves causes the mechanism of water vapour removal to be identical as in the case of the determination of the sorption isotherms. The choice of temperature for the drying process also proved to be an important parameter. Drying curve determination at 20 °C gives the best fit with the classical method. This level of drying temperature minimises processes hindering the removal of water vapour, such as surface cracking or initial swelling, etc. Further research is required to verify whether the proposed method will be applicable for heterogeneous samples such as food materials and products. 

## Figures and Tables

**Table 1:** Surface area of the analysed starches.

Code	Surface Area, SBET (m^2^/g)		
	Determined from Sorption Isotherms	Determined from the Drying Curves	
		“Wet” Samples	“Dry” Samples
CS	153.84 ± 7.39 ^a^	148.64 ± 24.09 ^a^	408.44 ± 112.11 ^b^
PS	146.20 ± 6.60 ^a^	114.96 ± 13.21 ^a^	318.30 ± 80.54 ^b^
RS	155.57 ± 7.02 ^a^	153.77 ± 22.23 ^a^	387.51 ± 91.33 ^b^
TS	157.84 ± 7.24 ^a^	143.70 ± 15.12 ^a^	374.16 ± 90.04 ^b^
WS	151.02 ± 6.91 ^a^	133.02 ± 13.74 ^a^	329.27 ± 61.24 ^b^
CCH	157.04 ± 9.57 ^b^	111.47 ± 11.18 ^a^	365.69 ± 66.40 ^c^
CCHg	154.30 ± 7.58 ^a^	106.45 ± 10.73 ^a^	407.13 ± 59.58 ^b^
CP	150.38 ± 6.91 ^a^	117.62 ± 18.30 ^a^	381.73 ± 84.13 ^b^
TP	150.95 ± 7.26 ^a^	190.40 ± 16.77 ^b^	386.94 ± 60.52 ^c^

The same letters indicate values that are not significantly different at α = 0.05.

**Table 2:** Starch specific surface area.

Code	Surface Area, S_BET_ (m^2^/g)		
	Determined from Sorption Isotherms		Determined from the Drying Curves
	Adsorption	Desorption	“Wet” Samples
CS	151.46 ± 7.61 ^a^	156.21 ± 7.85 ^a^	148.64 ± 24.09 ^a^
PS	145.65 ± 7.32 ^a^	146.74 ± 7.38 ^a^	114.96 ± 13.21 ^a^
RS	156.10 ± 7.85 ^a^	155.04 ± 7.79 ^a^	153.77 ± 22.23 ^a^
TS	156.53 ± 7.87 ^a^	159.15 ± 8.00 ^a^	143.70 ± 15.12 ^a^
WS	149.82 ± 7.53 ^a^	152.22 ± 7.65 ^a^	133.02 ± 13.74 ^a^
CCH	162.94 ± 8.19 ^b^	151.14 ± 7.60 ^b^	111.47 ± 11.18 ^a^
CCHg	151.51 ± 7.62 ^b^	157.10 ± 7.90 ^b^	106.45 ± 10.73 ^a^
CP	149.10 ± 7.49 ^a^	151.66 ± 7.62 ^a^	111.62 ± 18.30 ^a^
TP	153.31 ± 7.71 ^a^	148.60 ± 7.47 ^a^	190.40 ± 16.77 ^b^

The same letters indicate values that are not significantly different at α = 0.05.

**Table 3:** Comparison of specific surface area determined from adsorption isotherms vs. drying curves.

Surface Area, S_BET_ (m^2^/g)						
Code	Determined from Adsorption Isotherms	Determined from the Drying Curves for “Wet” Samples at Temperature				
		20 °C	40 °C	60 °C	80 °C	100 °C
CS	151.46 ± 7.61 ^b^	156.14 ± 10.10 ^b^	165.82 ± 7.96 ^b^	152.82 ± 7.68 ^b^	148.53 ± 7.47 ^b^	118.91 ± 5.98 ^a^
PS	145.66 ± 7.32 ^d^	146.32 ± 7.84 ^d^	126.95 ± 8.68 ^bc^	116.99 ± 7.74 ^b^	96.29 ± 4.84 ^a^	84.26 ± 4.23 ^a^
RS	156.51 ± 7.85 ^b^	155.13 ± 13.45 ^b^	191.71 ± 23.35 ^c^	175.13 ± 8.80 ^bc^	148.25 ± 7.45 ^b^	99.67 ± 5.01 ^a^
TS	156.53 ± 7.87 ^c^	159.19 ± 9.83 ^c^	159.93 ± 11.22 ^c^	150.25 ± 7.54 ^bc^	132.24 ± 6.65 ^ab^	114.16 ± 5.74 ^a^
WS	149.83 ± 7.53 ^c^	152.96 ± 7.49 ^c^	141.83 ± 7.54 ^bc^	137.32 ± 6.90 ^bc^	116.80 ± 5.87 ^ab^	121.23 ± 6.09 ^a^
CCH	162.94 ± 8.19 ^c^	151.32 ± 6.49 ^c^	111.64 ± 5.64 ^b^	105.98 ± 5.33 ^ab^	95.54 ± 4.80 ^ab^	94.02 ± 4.72 ^a^
CCHg	151.52 ± 7.62 ^c^	157.71 ± 5.97 ^c^	103.42 ± 6.55 ^b^	98.89 ± 4.97 ^ab^	90.11 ± 4.52 ^ab^	84.27 ± 4.23 ^a^
CP	149.01 ± 7.49 ^c^	151.81 ± 7.60 ^c^	123.35 ± 8.83 ^b^	115.62 ± 5.81 ^b^	108.77 ± 5.47 ^b^	89.61 ± 4.50 ^a^
TP	153.32 ± 7.71 ^b^	148.78 ± 18.02 ^b^	290.34 ± 14.41 ^c^	281.53 ± 14.15 ^c^	117.06 ± 5.88 ^a^	114.33 ± 5.74 ^a^

The same letters indicate values that are not significantly different at α = 0.05.

**Table 4:** Comparison of specific surface area determined from desorption isotherms vs. drying curves.

Surface Area, S_BET_ (m^2^/g)						
Code	Determined from Desorption Isotherms	Determined from the Drying Curves for “Wet” Samples at Temperature				
		20 °C	40 °C	60 °C	80 °C	100 °C
CS	156.21 ± 7.85 ^b^	156.14 ± 10.10 ^a^	165.82 ± 7.96 ^b^	152.82 ± 7.68 ^b^	148.53 ± 7.47 ^b^	118.91 ± 5.98 ^a^
PS	146.74 ± 7.38 ^c^	146.32 ± 7.84 ^c^	126.95 ± 8.68 ^b^	116.99 ± 7.74 ^b^	96.29 ± 4.84 ^a^	84.26 ± 4.23 ^a^
RS	155.04 ± 7.79 ^b^	155.13 ± 13.45 ^b^	191.71 ± 23.35 ^c^	175.13 ± 8.80 ^bc^	148.25 ± 7.45 ^b^	99.67 ± 5.01 ^a^
TS	159.15 ± 8.00 ^c^	159.19 ± 9.83 ^c^	159.93 ± 11.22 ^c^	150.25 ± 7.54 ^bc^	132.24 ± 6.65 ^ab^	114.16 ± 5.74 ^a^
WS	152.22 ± 7.65 ^c^	152.96 ± 7.49 ^c^	141.83 ± 7.54 ^c^	137.32 ± 6.90 ^bc^	116.80 ± 5.87 ^a^	121.23 ± 6.09 ^ab^
CCH	151.14 ± 7.60 ^c^	151.32 ± 6.49 ^c^	111.64 ± 5.64 ^b^	105.98 ± 5.33 ^ab^	95.54 ± 4.80 ^ab^	94.02 ± 4.72 ^a^
CCHg	157.10 ± 7.90 ^c^	157.71 ± 5.97 ^c^	103.42 ± 6.55 ^b^	98.89 ± 4.97 ^ab^	90.11 ± 4.52 ^ab^	84.27 ± 4.23 ^a^
CP	151.66 ± 7.62 ^c^	151.81 ± 7.60 ^c^	123.35 ± 8.83 ^b^	115.62 ± 5.81 ^b^	108.77 ± 5.47 _b_	89.61 ± 4.50 ^a^
TP	148.60 ± 7.47 ^b^	148.78 ± 18.02 ^b^	290.34 ± 14.41 ^c^	281.53 ± 14.15 ^c^	117.06 ± 5.88 ^a^	114.33 ± 5.74 ^a^

The same letters indicate values that are not significantly different at α = 0.05.

**Table 5:** Goodness of fit of the models.

Determined from Isotherms	Number of Homogeneous Groups from Nine Observations				
	20 °C	40 °C	60 °C	80 °C	100 °C
Adsorption	8	3	4	2	1
Desorption	8	3	4	2	1

**Table 6:** Characteristics of the material used in the research.

Starches	Native/Modification	Code	Producer
Maize	Native	CS	Sigma-Aldrich
Potato	Native	PS	ZPZ”Lublin
Rice	Native	RS	Sigma-Aldrich
Tapioca	Native	TS	TATE&LYLE
Wheat	Native	WS	Sigma-Aldrich
Resistamyl 347 (maize)	Chemical *	CCH	TATE&LYLE
Merigel 347 (maize)	Chemical *	CCHg	TATE&LYLE
Esentiale (maize)	Physical **	CP	TATE&LYLE
Bliss (tapioca)	Physical **	TP	TATE&LYLE

Detailed description of the modification [19,20] *; [21] **.

**Table 7:** Moisture content of starch samples.

Code	Moisture (g H_2_O/100 g)	
	“Dry” Samples	“Wet” Samples
CS	9.87 ± 1.55	20.75 ± 1.80
PS	10.29 ± 2.09	26.38 ± 1.62
RS	9.81 ± 1.57	21.33 ± 1.25
TS	10.36 ± 1.48	21.44 ± 4.59
WS	10.89 ± 0.9	22.02 ± 0.89
CCH	10.21 ± 1.07	27.27 ± 0.45
CCHg	10.31 ± 2.17	24.04 ± 0.94
CP	11.84 ± 1.48	26.04 ± 1.48
TP	10.69 ± 1.93	23.52 ± 1.42

## Data Availability

Not applicable.

## Sample Availability

All samples are available from the authors.
